# My sibling’s mental illness: An interpretative phenomenological analysis of experiences of having an adult sibling with a mental illness in semi-rural South Africa

**DOI:** 10.4102/sajpsychiatry.v27i0.1585

**Published:** 2021-05-31

**Authors:** Lisa Saville Young, Raylene Flannigan

**Affiliations:** 1 Department of Psychology, Faculty of Humanities, Rhodes University, Grahamstown, South Africa; 2 Department of Health, Fort England Psychiatric Hospital, Grahamstown, South Africa

**Keywords:** sibling, mental illness, experiences, semi-rural, South Africa, qualitative, interpretative phenomenological analysis (IPA)

## Abstract

**Background:**

When there is a lack of resources in the community to support deinstitutionalisation, the siblings of an individual with a mental illness are the ones who are the most affected and vulnerable. Nevertheless, sibling care work is still largely unacknowledged in the mental health sector in low- and middle-income countries.

**Aim:**

This article describes and interprets the lived experiences of ‘black’ isiXhosa-speaking individuals having a sibling with a mental illness, to shed light on how mental health professionals might support and sustain the involvement of individuals in the treatment and care of their sibling.

**Setting:**

The study was conducted in a semi-rural town in the Eastern Cape of South Africa.

**Methods:**

The study employed a qualitative research design using interpretative phenomenological analysis as the research method. Semi-structured interviews were conducted and analysed.

**Results:**

The findings present interview extracts which give voice to participants’ experiences of financial burden, social burden and stigma, and of engaging with psychiatric treatment while providing care for their mentally ill sibling. Findings also highlight the positive aspects of caring for a sibling with a mental illness.

**Conclusion:**

This study specifically highlights the gendered nature of care work and siblings’ increased understanding of mental illness by virtue of their relationship with their brother or sister, thereby possibly pointing to sibling relationships as valuable relational resources for challenging stigma. The study findings suggest that calls for greater cooperation between healing belief systems should include dialogue with western religious belief systems alongside traditional healing belief systems.

## Background

The purpose of this article is to describe the experiences of ‘black’^1^ isiXhosa^2^-speaking individuals in a semi-rural town in the Eastern Cape of South Africa, who have adult siblings with a serious mental illness, with a view to guide mental healthcare professionals to develop programmes and interventions for low- and middle-income (LAMI) countries, which support and sustain the involvement of individuals in the treatment and care of their sibling with a mental illness.

In 2011, Peterson and Lund argued that while in South Africa some progress has been made in decentralised care for severe mental disorders, there are insufficient resources to adequately support community-based services, resulting in the classic revolving door phenomenon.^1^ When there is a lack of resources in the community to support deinstitutionalisation, the siblings of an individual with a mental illness are the most affected and vulnerable.^2^ Because parents are ageing, research has begun to shift towards siblings as caregivers of their mentally ill brothers or sisters,^3,4,5^ highlighting the invisibility of siblings to mental health services.^6^

Nevertheless, we do know from research in the global North that both positive and negative experiences result from relationships with a sibling who has a mental illness.^7,8,9^ A recent study has also noted the role of cultural values in caregivers’ sense-making around these experiences.^10^ Yet still little is known about the experiences of sibling caregivers in LAMI countries such as South Africa, where cultural beliefs around mental illness continue to play a significant role in health-seeking behaviour.^11^ What is known is that caregivers of adults with mental illness in an African context express many difficulties with caring for their relatives’ physical and emotional needs, particularly with regard to financial burdens.^12,13^

Furthermore, a study conducted with caregivers of mental health service users found that many families did not understand the causes of the illness of their relative but wished to be educated as they believed that this would provide them with better strategies for caregiving.^13^ However, it is unclear to what extent the experiences of family caregivers more broadly are similar to or different from the experiences of the siblings in particular. This article aims to give voice to the experiences of adult siblings who care for their brothers or sisters in contexts of poverty and mental illness, in order to propose ways in which mental health practitioners can support the sibling caregiving role in South Africa.

## Methods

### Study design and objectives

Interpretative phenomenological analysis (IPA) was the chosen qualitative method for this study, because of its commitment to the examination of how people make sense of their major life experiences and, by extension, to give voice to these experiences.^14^ The IPA is grounded in Heideggerian phenomenology which argues that we only come to know reality through our own subjectivity as we are always a ‘person in context’. Thus, IPA aims to give insider’s accounts of subject matters, approaching phenomena with sensitivity and responsiveness in order that any subject matter be revealed on its own terms.^15^ The IPA also argues, however, that interpretation of this phenomena is always part of the description process. Therefore, in this study the objective was to describe participants’ interpretations of their experiences of their sibling relationships and the researchers’ interpretations of these experiences. The latter is marked by an epistemological openness,^15^ with a view to shed light on how mental health professionals might support and sustain the involvement of individuals in the treatment and care of their siblings.

### Setting

The study took place in Makhanda, a semi-rural town in the Eastern Cape of South Africa, which is characterised by high levels of poverty and unemployment and low educational levels. Nearly 40% of the population (39.4%) falls below the poverty line^16^ and about 13.2% of family households has no income.^17^

### Study population and sampling strategy

The study utilised purposive sampling in order to recruit a fairly homogenous sample group in line with IPA studies; participants had to be ‘black’, isiXhosa-speaking adults (over 18 years), living in Makhanda, Eastern Cape, South Africa, and self-identified as living with or caring for a brother or sister with a severe mental illness, that is, the sibling must have been hospitalised at some point because of the mental illness. In addition, snowball sampling was used to increase the probability of finding participants. The sample consisted of five participants as the emphasis is not on generalisability of findings but on a thick interpretative description of a small sample of participants. It is important to note that while all the participants consider themselves to have a brother or sister with a mental illness, not all the participants’ ‘brothers’ and ‘sisters’ are biological siblings. This socially constructed nature of sibling relationships (rather than biologically defined) is not unusual in ‘black’ South African families, particularly in the rural areas of South Africa where households and social relationships are frequently fluid and migratory.^18^ Social bonds are defined by who is dependent on whom and who takes the responsibility for whom in a context of extreme poverty.^19^ The characteristics of the participants are represented in the Table 1.

**TABLE 1:** Participant characteristics.

Pseudonyms	Age in years	Gender	Employment status	Nature of relationship	Sibling’s diagnosis	Religious identification	Number of household members
Phiwo	23	Female	Student	Uncle	Bipolar mood disorder	Christian	4
Phumla	49	Female	Unemployed	Sibling	Schizophrenia	Christian	3
Lutho	22	Female	Student	Uncle	Schizophrenia	Christian	4
Andile	22	Male	Learner	Sibling	Schizophrenia	Christian	5
Nomsa	31	Female	Student, Part-time	Sibling	Substance-induced psychosis that led to schizophrenia	Christian	3

Note: Pseudonyms are used throughout to protect the identities of the participants.

### Data collection

An interview guide was developed by the authors and consisted of open-ended questions in line with a semi-structured interviewing style recommended for IPA.^20^ The interviews were conducted in isiXhosa by the second author. Each interview was approximately 1 h-long and was audio recorded.

### Data analysis

The interviews were transcribed and translated into English by the second author prior to engaging with systematic qualitative analysis of this data with the aim of developing an organised, detailed, plausible and transparent account of the meaning of the data.^21^ Each transcript was subjected to line-by-line analysis (coding), with a focus on the experiential claims, concerns and understandings of each participant. The codes were then grouped into subordinate themes (emergent patterns) and then into master themes. Points of convergence and divergence, commonality and nuances, were then explored for every case and then subsequently across multiple cases. A dialogue developed between the researchers, the coded data and the researchers’ psychological knowledge from the literature review about what it might mean for participants to have a sibling diagnosed with a mental illness.

Yardley’s^22^ four principles for conducting quality qualitative research were applied throughout the study. Firstly, the researchers were careful to remain sensitive to the context in which the research was conducted facilitated by the fact that Both live and work in Makhanda. Moreover, the second author purposively conducted the interviewers with empathy and with a sensitivity to the possible power dynamics inherent in the interview exchange. Secondly, the researchers worked with commitment and rigor, immersing themselves in the available literature on the topic and the research method. Data collection and analysis were thoroughly and systematically conducted. Thirdly, transparency and coherence were attained by documenting all procedures throughout the study, including paying careful attention to researcher reflexivity. Finally, this article attests to the fourth principle, namely, the impact and importance of the research.

### Ethical considerations

Informed consent was gained from all the participants whose participation was voluntary. Anonymity and confidentiality of their data were guaranteed. The research was granted ethical approval by the appropriate ethics committee at Rhodes University.

## Results

Several themes emerged from the data, which are presented as follows: (1) experiencing the sibling as a burden post-diagnosis, (2) positive experiences emerging from the sibling’s mental illness and (3) experiences of the sibling’s treatment and the mental health system.

### Experiencing the sibling as a burden post-diagnosis

A common and dominant experience amongst participants was the significant financial strain faced by the participants having a sibling with a mental illness. The participants described feeling overwhelmed by the sense of financial responsibility that they were required to take on because of the loss of the sibling’s income:

‘I was very worried because she was very helpful at home, she used to buy furniture, buy clothes even for me you see. Because my mother didn’t get paid much, … So she was a breadwinner, I can say that. So I felt very sad, very sad because I knew that I would struggle this much.’ (Phumla, 49 years, female)

One participant described the sacrifice made by her as a result of the additional financial strain:

‘Always me, always me. I even ask myself sometimes, “Why God me?” … always, always. It also disturbs me. It’s a lot … And I also decided to leave the school. Oh man, shit! Everything is on me! Do you feel that? … Yho! It’s a burden.’ (Nomsa, 31 years, female)

For others, financial strain arose as a result of the conflict regarding the contribution of the disability grant:

‘Since I’m this kind of person who is responsible for all of them when they got sick and stuff. So now he is, he’s a grant payer, yes. Then on his day, nobody is taking his grant or his card what(so)ever. You will see him on the 1st or on the 31st or on the 20-something when the grant day is coming. He will start saying words like, “You won’t help me.” Saying to me, “You won’t help me,” “You will help your brothers”… blah blah … “I won’t get sick again”… something like that. Whereas I did not say anything. I just keep quiet. My dad said, “Just keep quiet. Leave him like that.” After a few days when the money is out, “Hello my sister. How are you?,” and then I say, “Hi, I’m fine thanks,” and so on, and stop there.’ (Nomsa, 31 years, female)

In the above extract, we see that Nomsa feels that it is unfair that she is financially responsible for her brother, because he does not provide his grant money to assist at home, becoming very elusive when the time draws closer to the date for his grant. There may be a gendered aspect to this, as earlier Phumla also describes her father not contributing financially to the household, despite being employed. Nomsa is conflicted as to how she should act upon her feelings because her father says that she should keep quiet; therefore, to maintain the peace she avoids expressing her true feelings. It is possible that her silence is also a function of her gendered position within a fairly traditional family.

In addition to the financial strain, the participants described experiencing many negative emotions related to the associated stigma of having a mentally ill sibling, particularly embarrassment, shame and fear:

‘Yho! It was very … I was feeling so like, I was not like myself because he (is) sometimes embarrassing you, us as a family. And then you feel those … and others were laughing. It was like he is doing it for fun. Sometimes he would go outside naked, you know those things.’ (Nomsa, 31 years, female)

Nomsa describes experiencing embarrassment as a result of her brother’s behaviour which she interprets as possibly intentional. She describes how this embarrassment stemmed from the laughter or ridicule of community members highlighting the stigma of mental illness.

Andile’s interview painted a similar picture of emotional strain largely because of stigmatisation of his mentally ill sibling by peers:

‘Mm for me I will say, having a brother with a mental disease, you face many difficulties. Because while I was growing up I didn’t understand. So the other kids were teasing him at the small age. So it wasn’t good at that time. It was bad because all the time when we see him, they would start singing songs that are based on him.’ (Andile, 22 years, male)

### Positive experiences emerging from the sibling’s diagnosis

Participants described positive changes in their own development as a result of having a relationship with their mentally ill sibling. For Andile this emerged in an increased sense of responsibility for his sibling’s well-being, leading to feeling closer to his brother:

‘Sometimes I am just being with him just to make him feel okay. Especially when I see that he is not in a good mood or good position, I like to just chill with him and take my guitar and play my guitar, I know he likes listening to music. And (I) play my guitar. So I would say his illness brought me close to him because as (his) brother, I always want to be better.’ (Andile, 22 years, male)

Phiwo’s sibling’s mental illness led to an increased sense of understanding of and patience with her brother’s unusual behaviour:

‘No it wasn’t affected. Maybe if ever I was shouting at him and I was saying, “Zola, do this!,” maybe it would have changed. But, even the things that he says about me sometimes I just choose to ignore him because I just (say to) myself, “No, because he’s got bipolar.” So I just understand.’ (Phiwo, 23 years, female)

A pattern of responses emerged from the data which pointed to participants’ experiences of having a greater understanding of mental illness as a result of their sibling’s diagnosis, either because of interacting with other people with mental illnesses, met through their sibling, or through experiencing less fear of mental illness because of their interactions with their sibling:

‘(It) makes me closer to other people who have this illness because almost all of his friends, they are like my friends now. Because even in the streets I holler at them now, “Hey brother! Hello.” And I stand and I chat and then I go. So it makes me closer to them.’ (Andile, 22 years, male)

‘It has helped me a lot because I now understand a person who has the same condition. It really has helped living with her as well. Now I’m not scared if I see someone like her. I also know now what I should do. I understand that she is also a human being and you shouldn’t shout when talking to her as though you’re talking to a mad person. You must address her as a normal human being.’ (Phumla, 24 years, female)

### Negotiating their sibling’s treatment and experiences of the mental health system

Most participants described their cultural and religious beliefs as significant in their experience of their sibling’s mental illness. Specifically, participants talked about how cultural expectations and their own spiritual beliefs informed their understanding of their sibling’s mental illness and in turn the help-seeking behaviour for their sibling. Some participants found negotiating these two different worldviews easier than others. Phumla describes going against the wishes of the extended family to take her sister to a traditional healer, opting instead to pray for her which she believes assisted in her healing:

‘They’re very happy now because they were my relatives. They were just advising me, “This woman is supposed to be a *sangoma* (traditional healer).” I said to them, “No! My mother tried all of these things but they never helped. So I’m going to do things on my own.” And I just started praying and nothing else … I see God worked there because I’m telling you I never took her to the witchdoctors. I just pray and she ended up stopping that swearing and going all over.’ (Phumla, 24 years, female)

Phumla describes drawing on her Christian belief and, therefore, rejecting cultural traditions, in seeking assistance for her sister. She does not mention medication as assisting with her sister’s symptoms subsiding, suggesting a primary understanding of mental illness as a spiritual illness:

‘Mother said to my father, “Maybe he needed something. Maybe like a traditional ceremony in order for him to be okay again.” But they (did) that ceremony but still there was no difference. Family members said that we should take him to the *sangoma* and we said that we did that and it didn’t help. It’s just a waste of money “cause we said he’s sick so he’s under treatment.” Yho! My belief? Yes, I do believe in those traditional things because we wanted my uncle to get healed but it didn’t help. They said we must slaughter a goat in order for him to be okay, but that didn’t help.’ (Lutho, 22 years, female)

Although Lutho perceives the traditional ceremonies as unhelpful in assisting with her sibling’s difficulties, she describes reluctance in giving up her traditional cultural beliefs. A distinction is made between mental illness as a biomedical problem which requires (psychiatric) treatment and mental illness as a spiritual problem which requires traditional ceremonies to appease the ancestors. It seems that for Phiwo the distinction between the two is difficult to hold on to. This tension between traditional and psychiatric approaches to treatment was prevalent in the participants’ narratives:

‘People said that if ever a person puts in those things, they might end up getting mad. So some are saying (that), “No, he is mad.” The doctor is saying, “It’s bipolar.” So we really do not know which is which. But I believe that it’s bipolar because when he’s eating his treatment he’s fine, you know. So that divided the family because of that … so we really don’t know which is which.’ (Phiwo, 23 years, female)

In the above extract, Phiwo describes how some of her family members told them that if a person has spirits inserted into them by a traditional healer to protect them (known as *izinyanya*), then those ‘things’ can also make the person ‘mad’. Here Phiwo seems to be saying that it was possibly the traditional approach that was the cause of her brother’s ‘madness’. The extract points to confusion and possibly conflict around the aetiology of her brother’s mental illness.

Overall participants seemed to draw a connection between their sibling’s psychiatric treatment and their improvement and spoke positively about their engagement with mental health service providers.

‘The support … Yho! … very … they were doing a very, very good role. I don’t know how to thank them because since then, it was only two or three times that he got sick. Since he is on treatment, I can say two times or three times.’ (Nomsa, 31 years, female)

The experience of feeling that the mental health service providers consulted with the participants regarding their sibling’s treatment varied. Lutho described mental health service providers giving careful explanations of the course of the illness:

‘Ja, they did explain to my parents and they explained it to us, that he would see things, hallucinate and hear voices. Ja so we would understand. But he would take his treatment and he would calm down a bit.’ (Lutho, 22 years, female)

But Nomsa described not receiving any information, despite feeling like she had multiple opportunities to receive this information:

‘Nope, I did go maybe twice or three times a day, I mean, a week but nothing they told us about the treatment.’ (Nomsa, 31 years, female)

The multiplicity of caregivers and geographical location were raised as possible reasons for the lack of information provided:

‘Maybe they told my mom, but by the time I took her, she was already in [*hospital*]. Oh no, I don’t know, but it’s an injection and some tablets. I meant to bring the tablets with.’ (Phumla, 24 years, female)

‘I wasn’t there. I wasn’t there because his doctor is in Uitenhage so I was only just told that these are his doctors. So I wasn’t there.’ (Phiwo, 23 years, female)

## Discussion

The participants in this study clearly contribute to the management of their siblings’ mental illness and have been affected by their siblings’ ill health, both negatively and positively. In line with much of the existing literature in contexts like these,^12,13,23^ the participants described experiencing financial strain as a result of their sibling’s mental illness.

Nevertheless, the phenomenological emphasis of this study uncovered the extent to which income is still controlled by men within the family and suggests that more provision needs to be made for the caring work and household maintenance that frequently falls on sisters and women.^17,24^

The impact of stigma on siblings is particularly evident in the findings of this study, supporting research that points to the high overall degree of perceived stigmatisation of mental illness in South Africa^25^ and other parts of Africa.^26^ The participants in this study reported remaining silent in the face of stigma within their communities, experiencing shame and embarrassment in line with the international findings.^27^ It is possible that these feelings are influenced by cultural beliefs which play into the stigma,^25^ as cultural beliefs of mental illness were at times linked to ‘spirits’ and ‘witchcraft’ as has been reported elsewhere.^28^

The participants of this study also described a number of positive experiences emerging from their relationship with their mentally ill sibling. These descriptions of aspects of fulfillment from the caregiver relationship are similar to those reported by caregivers in a Cape Town study.^29^ What emerged as different in this study was that many of the participants described the positive effects of a greater understanding of mental illness as a result of their siblings’ experiences, pointing to the possible value of sibling relationships in breaking down the barriers of stigmatisation of mental illness.

The participants in this study described negotiating two different belief systems, at times simultaneously, when seeking treatment of their siblings, supporting claims that individuals with severe mental disorders often utilise both westernised public healthcare facilities and traditional healers concurrently or sequentially.^1,30^ Indeed, many participants in this study described traditional healers as the first point of contact, supporting the findings by Mkize and Uys^11^; in many ways, this is unsurprising as the Xhosa culture, like many African cultures, considers the ancestors to be the guardians of family affairs, traditions, ethics and activities.^31,32^ It is evident through the results of the study that culture plays a vital role in the help-seeking behaviour. However, the findings significantly suggest that western religious beliefs also play a role in health-seeking behaviours alongside, and at times in conflict with, the traditional healing systems. Participants’ experiences of confusion and conflict as a result of negotiating with different systems of healing support claims made by Peterson and Lund^1^ that there are few examples of cooperation between different illness frameworks and highlight the problems resulting from this. Peterson and Lund^1^ call for the increased training of traditional healers to promote mental health literacy; we would add to this the need for increased training of religious leaders.

The current study suggests that caregiver information about the mental illness of their sibling was not forthcoming from mental health service providers, despite participants showing a desire to be informed or educated about their sibling’s mental illness. This finding is supported by Mavundla et al.^13^ and Cleary et al.^33^ Despite the lack of information provision, the participants in this study reported experiencing the mental health services positively, which suggests that in certain contexts there is a platform for building more empathic communication between mental health professionals and family carers. However, some of the participants reported not being involved in their siblings’ mental health treatment, suggesting continued invisibility of siblings as important sources of care. At times, this lack of involvement was a result of geographical distance or because of multiple caregivers’ participation, pointing to pragmatic challenges in involving siblings in mental health treatment.

A methodological limitation of the study is its small sample size; the findings cannot be generalised to the broader population. Nevertheless, the in-depth nature of the data collection and analysis which were facilitated by the small sample, meant that the findings could provide a rich description of experiences of siblings from a semi-rural context in South Africa.

## Conclusion

This study has drawn attention to the experiences of adult siblings as caregivers of their brothers or sisters with a mental illness in a semi-rural context in South Africa. Siblings’ experiences are in many ways similar to those reported by studies on family caregivers of individuals with mental illnesses including experiences of financial strain, experiences of stigmatisation, positive experiences of caregiving, confusion and conflict negotiating multiple belief systems when seeking help and both positive and negative experiences of mental health services. This study specifically highlights the gendered nature of care work which suggests that some men might retain control over finances despite sisters frequently bearing much of the burden of caregiving and home maintenance. The study also points to siblings’ increased understanding of mental illness by virtue of their relationship with their brother or sister and thereby possibly pointing to sibling relationships as valuable resources for challenging stigma. Finally, the study supports calls for greater cooperation between healing belief systems to promote cultural congruence and argues that this should include dialogue with western religious belief systems alongside traditional healing belief systems.

## Footnotes

1 The socially constructed nature of ‘race’ is acknowledged in this work and the researchers, thus, use single quotation marks to connote this signifier. The signifier is still globally considered relevant but also specifically in South Africa where racism continues to influence lived experiences.

2 IsiXhosa is an indigenous language spoken in the Eastern Cape of South Africa, in particular. While it predominantly refers to a language grouping, it is also considered a cultural grouping denoted by particular Xhosa rituals and customs.

## References

[1] Petersen I, Lund C. Mental health service delivery in South Africa from 2000 to 2010: One step forward, one step back. S Afr Med J. 2011;101(10):751–757. 22272856

[2] Friedrich RM, Lively S, Rubenstein LM. Siblings coping strategies and mental health services: A national study of siblings of persons with schizophrenia. Psychiatr Serv. 2008;3:261–267. 10.1176/ps.2008.59.3.261 18308906

[3] Hatfield A, Lefley HP. Future involvement of siblings in the lives of persons with mental illness. Community Ment Health J. 2005;41:327–338. 10.1007/s10597-005-5005-y 16131010

[4] Smith MJ, Greenberg JS, Seltzer M. Siblings of adults with schizophrenia: expectations about future caregiver roles. Am J Orthopsychiatry. 2007;77(1):29–37. 10.1037/0002-9432.77.1.29 17352582 PMC2396553

[5] Smith MJ, Greenberg JS. Factors contributing to the quality of sibling relationship with Schizophrenia. Psychiatr Serv. 2008;59(1):57–62. 10.1176/ps.2008.59.1.57 18182540 PMC2396577

[6] Sin J, Moone N, Harris, P. Siblings of individuals with first-episode psychosis: Understanding their experiences and needs. J Psychosoc Nurs. 2008;46(6): 34–38. 10.3928/02793695-20080601-11 18595457

[7] Barak D, Solomon Z. In the shadow of schizophrenia: A study of siblings’ perceptions. Isr J Psychiatry Relat Sci. 2005;42(4):234. 16618055

[8] Jewell TC. Impact of mental illness on well siblings: A sea of confusion. J NAMI. 2000;11(2):34–36.

[9] Tanaka K. Experiences of siblings of persons with major mental illness: Self development and support. Glob J Interdiscip Soc Sci J. 2001;5(11):76–79. 10.18848/1833-1882/CGP/v05i11/51939

[10] Han M, Diwan S, Sun K. Exploring caregiving-related experiences among Chinese American and European American family caregivers of persons with mental illness. Transcult Psychiatry. 2019;56(3):491–509. 1363461519827690. 30758257 10.1177/1363461519827690

[11] Mkize LP, Uys LR. Pathways to mental health in Kwazulu Natal. Curationis. 2004;27(3):62–71. 10.4102/curationis.v27i3.1001 15777031

[12] Lund C, De Silva M, Plagerson S, et al. Poverty and mental disorders: Breaking the cycle in low-income and middle-income countries. Lancet. 2011;378(9801):1502–1514. 10.1016/S0140-6736(11)60754-X 22008425

[13] Mavundla TR, Toth F, Mphelane ML. Caregiver experience in mental illness: A perspective from a rural community in South Africa. Int J Ment Health Nurs. 2009;18(5):356–367. 10.1111/j.1447-0349.2009.00624.x 19740145

[14] Smith JA, Flowers P, Larkin M. Interpretative phenomenological analysis: Theory, method and research. London: Sage; 2009

[15] Larkin M, Watts S, Clifton E. Giving voice and making sense in interpretative phenomenological analysis. Qual Res Psychol. 2006;3(2):102–120. 10.1191/1478088706qp062oa

[16] Socioeconomic Specialist Report. Belmont Valley and existing Grahamstown Golf course development [homepage on the Internet]. In J Roodt. Grahamstown; n.d [cited 2018 May 11]. Available from: https://sahris.sahra.org.za/sites/default/files/additionaldocs/6.%20Socio-Economic%20Specialist%20Report_0.pdf

[17] Statistics South Africa. Poverty trends in South Africa: An examination of absolute poverty between 2006 and 2011 (Report No. 03-10-06) [homepage on the Internet]. 2014 [cited 2018 May 11]. Available from: https://www.statssa.gov.za/publications/Report-03-10-06/Report-03-10-062015.pdf

[18] Bozalek V. Contextualising caring in Black South African families. Soc Politics. 1999;6(1):85–99. 10.1093/sp/6.1.85

[19] Hosegood V, Benzler J, Solarsh GC. Population mobility and household dynamics in rural South Africa: Implications for demographic and health research. South Afr J Demog 2005;10(1/2):43–68.

[20] Willig C. Introducing qualitative research in psychology. New York: McGraw-Hill Education; 2013.

[21] Larkin M, Thompson AR. Interpretative phenomenological analysis in mental health and psychotherapy research. In Harper D, Thompson AR, editors. Qualitative research methods in mental health and psychotherapy: A guide for students and practitioners. Chichester: John Wiley & Sons, Ltd; 2012; p. 99–116.

[22] Yardley L. Dilemmas in qualitative health research. Psychol Health. 2000;15(2):215–228. 10.1080/08870440008400302

[23] Addo R, Agyemang SA, Tozan Y, Nonvignon J. Economic burden of caregiving for persons with severe mental illness in sub-Saharan Africa: A systematic review. PLoS One. 2018;13(8):e0199830. 10.1371/journal.pone.0199830 30092073 PMC6084810

[24] Bhan N, Rao N, Raj A. Gender differences in the associations between informal caregiving and wellbeing in low-and middle-income countries. J Women Health. 2020;29(10):1328–1338. 10.1089/jwh.2019.7769 PMC7583325 32159418

[25] Botha UA, Koen L, Niehaus DJ. Perceptions of a South African schizophrenia population with regards to community attitudes towards their illness. Soc Psychiatry Psychiatr Epidemiol. 2006;41(8):619–623. 10.1007/s00127-006-0071-1 16733630

[26] Ae-Ngibise KA, Doku VCK, Asante KP, Owusu-Agyei S. The experience of caregivers of people living with serious mental disorders: A study from rural Ghana. Glob Health Action. 2015;8(1):26957. 10.3402/gha.v8.26957 25967587 PMC4429259

[27] Larson JE, Corrigan P. The stigma of families with mental illness. Acad Psychiatry. 2008;32:87–91. 10.1176/appi.ap.32.2.87 18349326

[28] Monyaluoe M, Mvandaba M, Plessis ED, Koen MP. Experiences of families living with a mentally ill family member. J Psychiatr [serial online]. 2004 [cited n.d.];17:131. Available from: https://www.longdom.org/open-access/experiences_of_families_living_with_a_mentally_ill_family_member_psychiatry_131.pdf

[29] Sibeko G, Milligan PD, Temmingh H, Lund C, Stein DJ, Mall S. Caregiving for mental health service users: A study exploring the perceptions of mental health service users and their caregivers in Cape Town, South Africa. Int J Soc Psychiatry. 2016;62(6):512–521. 10.1177/0020764016651458 27282176

[30] Debrah AB, Buabeng KO, Donnir G, Akwo Kretchy I. A caregiver perspective of complementary and alternative medicine use among patients with schizophrenia and bipolar disorders. Int J Ment Health. 2018;47(4):298–310. 10.1080/00207411.2018.1546097

[31] Bojuwoye O. Traditional healing practices in Southern Africa: Ancestral spirits, ritual ceremonies, and holistic healing. In Moodley R, West E, editors. Integrating traditional healing practices into counseling and psychotherapy. Thousand Oaks, CA: Sage, 2005; p. 61–72.

[32] Vontress CE. Animism: Foundation of traditional healing in sub-Saharan Africa. In Moodley R, West E, editors. Integrating traditional healing practices into counselling and psychotherapy. Thousand Oaks, CA: Sage, 2005; p. 124–137.

[33] Cleary M, West S, Hunt GE, McLean L, Kornhaber R. A qualitative systematic review of caregivers’ experiences of caring for family diagnosed with schizophrenia. Issues Ment Health Nurs. 2020;41(8):667–683. 10.1080/01612840.2019.1710012 32255401

